# Rabies Inoculation

**Published:** 1884-09

**Authors:** 


### Rabies Inoculation.

It is now about four years since Pasteur commenced his experiments and researches into the nature of hydrophobia, the results of which have been recently given to the public. Although the profession and scientists generally may not be very sanguine as to the grand results which this distinguished *savant* claims, yet enough has been advanced to warrant the French government in appointing a commission of scientific men of indisputable authority to investigate the matter and to test the value of the interesting experiments instituted by Pasteur. The names of Vulpian, Villemin, Bert and Bouley are a sufficient guarantee of the character and reliability of the proposed enquiry. Pasteur, in the course of his experiments, hit upon the expedient of inoculating the brain of the animal with the virus of rabies. The skull is trephined with a small instrument and the virus introduced. By this method the action of the virus is much hastened, the effects being manifest in a few days, instead of from twelve to fourteen days. In fact, Pasteur thinks he has in this way demonstrated that rabies is a malady of the brain. In the course of his experiments he found that the virus, after having passed through three monkeys in succession, becomes so attenuated that its introduction into a dog is harmless. But when the virus is passed through a rabbit and guinea pig in like manner, it increases in virulence, becoming more virulent than the virus of the rabid dog. The plan proposed is to take the virus from a rabbit dying after inoculation, and inoculate this successively in other rabbits, and finally in the dog, which is thus rendered refractory to the rabies.

The test experiments proposed by Pasteur consist, first, in causing twenty unprotected dogs and twenty "vaccinated" dogs (presumably protected thereby from the poison) to be bitten by dogs in a rabid state; and, second, in artificially inoculating with the virus of rabies two other sets of twenty dogs, respectively vaccinated and unvaccinated. "The twenty vaccinated dogs," says Pasteur, "will resist the poison, and the other twenty will all die of madness."

The importance of this discovery, if true, cannot be overestimated, but we must not be too ready to express unqualified approval and endorsement of Pasteur's views. It will be observed that he uses, contrary to what one would have supposed, the virus from rabbits, and not the attenuated virus from monkeys. Furthermore, he does not propose to apply the virus for the protection of human beings, although we have read in the press that persons applied to him for inoculation. The experiments, so far, do not seem to us convincing, and we wait with considerably curiosity, mingled with not a little anxiety, the report of the commission. The result of these trials can hardly fail to be largely decisive of the question one way or the other, and will be an unequivocal illustration of the value of experimental pathology. Meantime, we agree with the man who said that the best way to prevent hydrophobia was "to shoot the dog before he went mad."—*Canada Lancet.*

